# ADGRL3 (LPHN3) variants predict substance use disorder

**DOI:** 10.1038/s41398-019-0396-7

**Published:** 2019-01-29

**Authors:** Mauricio Arcos-Burgos, Jorge I. Vélez, Ariel F. Martinez, Marta Ribasés, Josep A. Ramos-Quiroga, Cristina Sánchez-Mora, Vanesa Richarte, Carlos Roncero, Bru Cormand, Noelia Fernández-Castillo, Miguel Casas, Francisco Lopera, David A. Pineda, Juan D. Palacio, Johan E. Acosta-López, Martha L. Cervantes-Henriquez, Manuel G. Sánchez-Rojas, Pedro J. Puentes-Rozo, Brooke S. G. Molina, MTA Cooperative Group, Margaret T. Boden, Deeann Wallis, Brett Lidbury, Saul Newman, Simon Easteal, James Swanson, Hardip Patel, Nora Volkow, Maria T. Acosta, Francisco X. Castellanos, Jose de Leon, Claudio A. Mastronardi, Maximilian Muenke

**Affiliations:** 1 Medical Genetics Branch, National Human Genome Research Institute, National Institutes of Health, Bethesda, MD USA; 2 INPAC Research Group, Fundación Universitaria Sanitas, Bogotá, Colombia; 3 Instituto de Investigaciones Médicas (IIM), Facultad de Medicina, Universidad de Antioquia, Medellín, Colombia; 4 Universidad del Norte, Barranquilla, Colombia; 5 Psychiatric Genetics Unit, Group of Psychiatry, Mental Health and Addiction, Vall d’Hebron Research Institute (VHIR), Universitat Autònoma de Barcelona, Barcelona, Spain; 6 Department of Psychiatry, Hospital Universitari Vall d’Hebron, Barcelona, Spain; 7 Biomedical Network Research Centre on Mental Health (CIBERSAM), Barcelona, Spain; 8 Department of Psychiatry and Legal Medicine, Universitat Autònoma de Barcelona, Barcelona, Spain; 9 Addiction and Dual Diagnosis Unit, Departament of Psychiatry, Hospital Universitari Vall d’Hebron-Public Health Agency, Barcelona, Spain; 10 Department of Genetics, Microbiology and Statistics, University of Barcelona, Barcelona, CAT Spain; 11 Centro de Investigación Biomédica en Red de Enfermedades Raras (CIBERER), Instituto de Salud Carlos III, Madrid, Spain; 12 Institut de Biomedicina de la Universitat de Barcelona (IBUB), Barcelona, CAT Spain; 13 Institut de Recerca Sant Joan de Déu (IRSJD), Esplugues, CAT Spain; 14 Neuroscience Research Group, Universidad de Antioquia, Medellín, Colombia; 15 Grupo de Neurociencias del Caribe, Unidad de Neurociencias Cognitivas, Universidad Simón Bolívar, Barranquilla, Colombia; 16 Grupo de Neurociencias del Caribe, Universidad del Atlántico, Barranquilla, Colombia; 17 Departments of Psychiatry and Psychology, University of Pittsburg, Pittsburg, PA USA; 18 University of Kentucky Mental Health Research Center at Eastern State Hospital, Lexington, KY USA; 19 Department of Biochemistry and Biophysics, Texas A&M University, College Station, TX USA; 20 National Center for Indigenous Genomics, Genome Biology Department, John Curtin School of Medical Research, ANU College of Medicine, Biology and Environment, The Australian National University, Canberra, ACT Australia; 21 Department of Psychiatry, Florida International University, Miami, FL USA; 22 Child Development Center, University of California at Irvine, Irvine, CA USA; 23 Genome Discovery Unit, Genome Biology Department, John Curtin School of Medical Research, ANU College of Medicine, Biology and Environment, The Australian National University, Canberra, ACT Australia; 24 Office of the Director, National Institute on Drug Abuse, National Institutes of Health, Rockville, MD USA; 25 Department of Child and Adolescent Psychiatry, Hassenfeld Children’s Hospital at NYU Langone, New York, NY USA; 26 Nathan Kline Institute for Psychiatric Research, Orangeburg, NY USA; 27 Center for Research in Genetics and Genomics, Institute of Translational Medicine, School of Medicine and Health Sciences, Universidad del Rosario, Bogotá, Colombia

## Abstract

Genetic factors are strongly implicated in the susceptibility to develop externalizing syndromes such as attention-deficit/hyperactivity disorder (ADHD), oppositional defiant disorder, conduct disorder, and substance use disorder (SUD). Variants in the *ADGRL3* (*LPHN3*) gene predispose to ADHD and predict ADHD severity, disruptive behaviors comorbidity, long-term outcome, and response to treatment. In this study, we investigated whether variants within *ADGRL3* are associated with SUD, a disorder that is frequently co-morbid with ADHD. Using family-based, case-control, and longitudinal samples from disparate regions of the world (*n* = 2698), recruited either for clinical, genetic epidemiological or pharmacogenomic studies of ADHD, we assembled recursive-partitioning frameworks (classification tree analyses) with clinical, demographic, and *ADGRL3* genetic information to predict SUD susceptibility. Our results indicate that SUD can be efficiently and robustly predicted in ADHD participants. The genetic models used remained highly efficient in predicting SUD in a large sample of individuals with severe SUD from a psychiatric institution that were not ascertained on the basis of ADHD diagnosis, thus identifying *ADGRL3* as a risk gene for SUD. Recursive-partitioning analyses revealed that rs4860437 was the predominant predictive variant. This new methodological approach offers novel insights into higher order predictive interactions and offers a unique opportunity for translational application in the clinical assessment of patients at high risk for SUD.

## Introduction

Substance use disorders (SUD) and addiction represent a global public health problem of substantial socioeconomic implications^1,2^. In 2010, 147.5 million cases of alcohol and drug abuse were reported (Whiteford et al., 2015), and SUD prevalence is expected to increase over time. Genetic factors have been implicated in SUD etiology, with genes involved in the regulation of several neurobiological systems (including dopaminergic and glutamatergic) found to be important (for a review see Prom-Wormley et al., 2017^3^). However, limitations intrinsic to most genetic epidemiological studies support the search for additional risk genes.

Attention-deficit/hyperactivity disorder (ADHD), the most common neurodevelopmental behavioral disorder^4,5^, is frequently co-morbid with disruptive behaviors such as oppositional defiant disorder (ODD), conduct disorder (CD), and SUD^6,7^. The close association between ADHD and disruptive behaviors is summarized by longitudinal observations in ADHD cohorts^6,8,9^. Children diagnosed with ADHD monitored during the transition into adolescence exhibit higher rates of alcohol, tobacco, and psychoactive drug use than control groups of children without ADHD^10,11^. It has been estimated that the lifetime risk for SUD is ~50% in subjects with childhood ADHD persisting into adulthood^12,13^. Reciprocally, the prevalence of ADHD is high in adolescents with SUD^9,14,15^ and the presence of an ADHD diagnosis affects SUD prognosis, with ADHD being associated with both earlier and more frequent alcohol-related relapses^16^ and lower likelihood of cannabis-dependence treatment completion^17^.

Strong evidence from family, twin, and genome-wide linkage and association studies suggests that genetic factors play a crucial role in shaping the susceptibility to both ADHD and SUD^18–21^. During the last 15 years, we have collected families clustering individuals affected with ADHD and disruptive behaviors from disparate regions around the world^6,18,22,23^. Although the prevalence of ADHD co-morbid with disruptive behaviors is variable across populations, we found a higher frequency of CD, ODD, and SUD (mainly nicotine dependence and alcohol abuse) in ADHD individuals than in unaffected relatives^6,22,24^. Using genome-wide data from extended multigenerational families, we found evidence of linkage of ADHD to markers in chromosomes 4q13.2, 5q33.3, 8q11.23, 11q22, and 17p11^25^, and co-segregation of ADHD and disruptive behaviors with loci at 2p21-22.3, 4q13.2, 5p13.1-p13.3, 8q24, 8q15, 11q22, 12p11.23-13.3, and 14q21.1-22.2^8^. Fine mapping of the 4q13.2 region identified variants in the adhesion G-protein-coupled receptor L3 gene *(ADGRL3*, also known as latrophilin 3 or *LPHN3)* that predispose to ADHD^22,24,26–30^.

Characterization of the association between ADHD and *ADGRL3* has provided key information to better predict the severity of ADHD, the long-term outcome, the patterns of brain metabolism, and the response to stimulant medication^24,27,29,31–34^. To the best of our knowledge, *ADGRL3* linkage and association results represent some of the most robustly replicated genetic and pharmacogenetic findings in ADHD genetic research. While *ADGRL3* has also shown association with disruptive behaviors in the context of ADHD^18,24,35^, a direct link to SUD has not been systematically investigated. In this manuscript we tested the hypothesis that ADHD risk variants harbored at the *ADGRL3* locus interact with clinical, demographic, and environmental variables associated with SUD.

## Subjects and methods

### Subjects

We used independent populations from disparate regions of the world (*n* = 2698) ascertained through patients affected with ADHD co-morbid with disruptive behaviors (Paisa, Spanish and MTA samples) or SUD (Spanish and Kentucky samples).

### Paisa sample

This population isolate is unique in that it was used to identify ADHD susceptibility genes by linkage and association strategies. Detailed clinical and demographic information on this sample has been published elsewhere^23,25,29^. The sample consists of 1176 people (adults, adolescents, and children), mean age 28 ± 17 years, ascertained from 18 extended multigenerational and 136 nuclear Paisa families inhabiting the Medellin metropolitan area in the State of Antioquia, Colombia. Initial coded pedigrees were obtained through a fixed sampling scheme from a parent or grandparent of an index proband after having collected written informed consent from all subjects or their parent/guardian, as approved by the University of Antioquia and the NIH Ethics Committees, and in accordance with the Helsinki Declaration. Patients were recruited under NHGRI protocol 00-HG-0058 (NCT00046059).

Exclusion criteria for ADHD participants were IQ < 80, or any autistic or psychotic disorders. Parents underwent a full psychiatric structured interview regarding their offspring (Diagnostic Interview for Children and Adolescents—Revised—Parents version (DICA-IV-P, Spanish version translated with permission from Dr. Wendy Reich (Washington University, St. Louis). All adult participants were assessed using the Composite International Diagnostic Interview (CIDI), as well as the Disruptive Behavior Disorders module from the DICA-IV-P modified for retrospective use. The interview was conducted by a “blind” rater (either a psychologist, a neuropsychologist, or a psychiatrist) at the Neurosciences Clinic of the University of Antioquia, or during home visits. ADHD status was defined by the best estimate method. Specific information regarding clinical diagnoses and co-morbid disruptive disorders, affective disorders, anxiety, and substance use has been published elsewhere^3^.

From the 1176 individuals in this cohort, only founder members were included in analyses (*n* = 472). This was done to avoid kinship relatedness bias and to exclude children and adolescents, as they may have not been exposed to substances of abuse yet. Of these 472 individuals, 17% (*n* = 79) fulfilled criteria for ADHD, 17% (*n* = 78) for ODD, 18% (*n* = 84) for CD, 22% nicotine dependence (*n* = 102), 27% alcohol dependence (*n* = 124), 3% drug dependence (*n* = 12), 37% social/simple phobia (*n* = 156), 13% any other anxiety disorder (*n* = 58), and 25% major depressive disorder (*n* = 117) (Table 1).

**Table 1:** A concise description of the cohorts’ principal demographic and clinical data.

	Paisa sample		Spanish sample		MTA sample		Kentucky sample	
	*n*	%	*n*	%	*n*	%	*n*	%
Sex								
Males	231	49%	1193	72%	287	76%	285	53%
Females	241	51%	454	28%	89	24%	248	47%
Total	472	100%	1647	100%	376	100%	533	
ADHD								
Affected	79	17%	670	41%	140	37%	^*a*^	^*a*^
Unaffected	249	53%	486	29%	236	63%	^*a*^	^*a*^
Unknown	144	30%	491	30%	376	100%	^*a*^	^*a*^
ODD								
Affected	78	17%	81	5%	^*a*^	^*a*^	^*a*^	^*a*^
Unaffected	250	53%	391	24%	^*a*^	^*a*^	^*a*^	^*a*^
Unknown	144	30%	1175	71%	^*a*^	^*a*^	^*a*^	^*a*^
CD								
Affected	84	18%	102	6%	^*a*^	^*a*^	^*a*^	^*a*^
Unaffected	244	52%	357	22%	^*a*^	^*a*^	^*a*^	^*a*^
Unknown	144	30%	1188	72%	^*a*^	^*a*^	^*a*^	^*a*^
Nicotine								
Affected	102	22%	646	39%	97	26%	372	70%
Unaffected	226	48%	613	37%	40	11%	161	30%
Unknown	144	30%	388	24%	239	63%	0	0%
Alcohol								
Affected	124	27%	396	24%	120	32%	342	64%
Unaffected	204	43%	637	39%	106	28%	191	36%
Unknown	144	30%	614	37%	150	40%	0	0%
Cannabis								
Affected	^*a*^	^*a*^	^*a*^	^*a*^	94	25%	^*a*^	^*a*^
Unaffected	^*a*^	^*a*^	^*a*^	^*a*^	71	19%	^*a*^	^*a*^
Unknown	^*a*^	^*a*^	^*a*^	^*a*^	211	56%	^*a*^	^*a*^
Other drugs								
Affected	12	3%	^*a*^	^*a*^	^*a*^	^*a*^	147	28%
Unaffected	197	56%	^*a*^	^*a*^	^*a*^	^*a*^	386	72%
Unknown	263	41%	^*a*^	^*a*^	^*a*^	^*a*^	0	0%
SUD								
Affected	^*a*^	^*a*^	768	47%	^*a*^	^*a*^	452	85%
Unaffected	^*a*^	^*a*^	879	53%	^*a*^	^*a*^	81	15%
Unknown	^*a*^	^*a*^	0	0%	^*a*^	^*a*^	^*a*^	^*a*^
Phobias								
Affected	156	37%	50	3%	^*a*^	^*a*^	^*a*^	^*a*^
Unaffected	172	33%	584	36%	^*a*^	^*a*^	^*a*^	^*a*^
Unknown	144	30%	1013	61%	^*a*^	^*a*^	^*a*^	^*a*^
Anxiety								
Affected	58	13%	107	6%	^*a*^	^*a*^	^*a*^	^*a*^
Unaffected	270	57%	818	50%	^*a*^	^*a*^	^*a*^	^*a*^
Unknown	144	30%	722	44%	^*a*^	^*a*^	^*a*^	^*a*^
Depression								
Affected	117	25%	143	9%	^*a*^	^*a*^	^*a*^	^*a*^
Unaffected	211	45%	490	30%	^*a*^	^*a*^	^*a*^	^*a*^
Unknown	144	30%	1014	61%	^*a*^	^*a*^	^*a*^	^*a*^
Mood								
Affected	^*a*^	^*a*^	^*a*^	^*a*^	^*a*^	^*a*^	157	29%
Unaffected	^*a*^	^*a*^	^*a*^	^*a*^	^*a*^	^*a*^	376	71%
Unknown	^*a*^	^*a*^	^*a*^	^*a*^	^*a*^	^*a*^	0	0%
Schizophrenia								
Affected	^*a*^	^*a*^	^*a*^	^*a*^	^*a*^	^*a*^	253	47%
Unaffected	^*a*^	^*a*^	^*a*^	^*a*^	^*a*^	^*a*^	280	53%
Unknown	^*a*^	^*a*^	^*a*^	^*a*^	^*a*^	^*a*^	0	0%

For the Paisa cohort, only information for founder members (adults) used in the ARPA-based predictive model for SUD is shown. See Methods section for more details

*ADHD* attention-deficit/hyperactivity disorder, *CD* conduct disorder, *ODD* oppositional defiant disorder, *SUD* substance use disorders

^*a*^Data not available

### Spanish sample

The ADHD sample consisted of 670 adult ADHD patients, mean age 33 ± 10 years, 69% males (*n* = 461), recruited and evaluated at the Psychiatry Department of the Hospital Universitari Vall d’Hebron (Barcelona, Spain) according to DSM-IV TR criteria. ADHD diagnosis was based on the Spanish version of the Conners Adult ADHD Diagnostic Interview for DSM-IV (CAADID)^36^. Comorbidity was assessed by Structured Clinical Interview for DSM-IV Axis I and Axis II Disorders (SCID-I and SCID-II). ODD during childhood and adolescence was retrospectively evaluated with the Schedule for Affective Disorders and Schizophrenia (SADS) for School-Age Children, Present and Lifetime Version (K-SADS). Thirty-nine percent of ADHD patients (*n* = 263) fulfilled diagnostic criteria for SUD, 21% for disruptive behavior disorders (CD and/or ODD; *n* = 142), 21% for depression (*n* = 143), 13% for anxiety (*n* = 89), and 8% for phobias (*n* = 50). The level of impairment was measured with the Clinical Global Impression (CGI) included in the CAADID Part II and the Sheehan Disability Inventory. Exclusion criteria for ADHD patients were IQ < 80; pervasive developmental disorders; schizophrenia or other psychotic disorders; presence of mood, anxiety or personality disorders that might explain ADHD symptoms; birth weight ≤ 1.5 kg; and other neurological or systemic disorders that might explain ADHD symptoms.

The SUD sample consisted of 494 adults (mean age 37 ± 9 years and 76% males, *n* = 376) recruited and evaluated at the Addiction and Dual Diagnosis Unit of the Psychiatry Department at the Hospital Universitari Vall d’Hebron with the Structured Clinical Interview for DSM-IV Axis I Disorders (SCID-I). All patients fulfilled DSM-IV criteria for drug dependence beyond nicotine dependence. None were evaluated for ADHD.

The control sample consisted of 483 blood donors (mean age 42 ± 20 years, 74% males) in which DSM-IV lifetime ADHD symptomatology was excluded under the following criteria: (1) not having been diagnosed with ADHD and (2) answering negatively to the lifetime presence of the following DSM-IV ADHD symptoms: (a) often has trouble keeping attention on tasks, (b) often loses things needed for tasks, (c) often fidgets with hands or feet or squirms in seat, and (d) often gets up from seat when remaining in seat is expected. Individuals affected with SUD were excluded from this sample. None of them had self-administered drugs intravenously. It is important to mention that the exposure criterion was not applied; therefore, this set cannot be classified as “pure” controls.

All patients and controls were Spanish of Caucasian descent. This study was approved by the ethics committee of the Hospital Universitari Vall d’Hebron and informed consent was obtained from all subjects in accordance with the Helsinki Declaration.

### MTA sample

The Multimodal Treatment Study of Children with ADHD (MTA) was designed to evaluate the relative efficacy of treatments for childhood ADHD, combined subtype, in a 14-month randomized controlled trial of 579 children assigned to four treatment groups: medication management, behavior modification, their combination, and treatment as usual in community care. After the 14-month treatment-by-protocol phase, the MTA continued as a naturalistic follow-up in which self-selected use of psychoactive medication was monitored. A local normative comparison group of 289 randomly selected classmates group-matched for grade and sex was added when the ADHD participants were between 9–12 years of age. The outcomes in childhood (14, 24, and 36 months after baseline), and adolescence (6 and 8 years after baseline) and into adulthood (12, 14, and 16 years after baseline) have been reported^10,11,37–43^. Substance use was assessed with a child/adolescent-reported questionnaire adapted for the MTA^11,43^. The measure included items for lifetime and current (previous 6 months) use of alcohol, cigarettes, tobacco, cannabis, and other recreational drugs. Also included were items for non-prescribed use or misuse of psychoactive medications, including stimulants. The measure was modeled after similar substance use measures in longitudinal or national survey studies of alcohol and other drug use that also rely on confidential youth self-report as the best source of data^44,45^. A National Institutes of Health (NIH) Certificate of Confidentiality further strengthened the assurance of privacy. Substance use was coded positive if any of the following behaviors, selected after examining distributions, were endorsed as occurring in the participant’s lifetime up to 8 years post-baseline: (1) alcohol consumption (more than just a sip) more than five times or drunk at least once; (2) cigarette smoking or tobacco chewing more than a few times; (3) cannabis use more than once; or (4) use of inhalants, hallucinogens, cocaine, or any of amphetamines/stimulants, barbiturates/sedatives, and opioids/narcotics without a prescription or misused a prescription (used in greater quantity or more often than prescribed). Each of the four types of substances, as well as daily use of tobacco and the number of substance use classes endorsed (0, 1, 2, or more), were explored in secondary analyses.

DSM-IV abuse or dependence was based on a positive parent or child report with the Diagnostic Interview Schedule for Children version 2.3/3.0 (DISC)^46^ at the 6- and 8-year follow-up assessments. The DISC includes both lifetime and past year diagnoses. The Diagnostic Interview Schedule-IV^47^ was used at the 8-year follow-up for 18 + year-olds (*n* = 111). SUD was defined as the lifetime presence of any abuse or dependence (excluding tobacco dependence, due to differences in the meaning of abuse/dependence for tobacco versus other substances).

Additional analyses explored SUD for alcohol, tobacco, and cannabis/other drugs (recreational or misused prescription medications) separately^10^. All patients in this study provided informed written consent as approved by the NIH Ethics Committee.

### Kentucky sample

A sample of 560 inpatients and outpatients with severe SUD from Central Kentucky psychiatric facilities was collected during a pharmacogenetics investigation^48^. Patient interviews and medical record information (including urine drug screens and substance abuse counselor notes) were used by the research nurse to assess the Clinician Rating of Alcohol (CRAUD) and Drug Use Disorder (CRDUD)^49,50^ that provides a score from 1 = abstinence (not used in the assessed period) to 5 = severe dependence. Scores of 3 and higher are pathological and were considered positive in our analyses. All drugs were combined into one rating^48^. Descriptions of the training provided to research nurses to assess the CRAUD and CRDUD were published elsewhere^48,51^.

DNA was available from 533 of 560 study subjects. Of the 533 subjects with available DNA, 53% (*n* = 285) were male, 82% (*n* = 436) were Caucasian, 16% (*n* = 87) were African American, and 2% (*n* = 10) were from other ethnicities. Additional clinical information for this sample has been described elsewhere^48,51^ and included: (1) clinical diagnosis obtained from medical records, (2) prior psychiatric history, (3) history of daily smoking, (4) reviews of current and psychiatric medication use, and (5) body mass index (Supplemental Table 2). All participants in the Kentucky study provided informed written consent as approved by the University of Kentucky IRB.

### Genotyping

DNA was extracted from whole blood (Paisa, Spanish and MTA sample) or buccal swabs (Kentucky sample) using standard protocols. The Paisa sample was genotyped using the service provided by Illumina (San Diego, CA). The Spanish, MTA, and Kentucky samples were genotyped for select variants using pre-designed TaqMan^®^ SNP genotyping assays (Thermo Fisher Scientific, Waltham, MA). Allelic discrimination real-time PCR reactions were performed in a 384-well plate format for each individual sample according to the manufacturer’s instructions. Briefly, 20 ng of genomic DNA were mixed with 2.5 μL of 2X TaqMan Universal PCR Master Mix and 0.25 μL of 20X SNP Genotyping Assay in a total volume of 5 μL per reaction. Assays were run in an ABI 7900HT Fast Real-Time PCR System (Thermo Fisher Scientific). Allele calling was made by end-point fluorescent signal analysis using the ABI’s SDS2.3 software. In addition, we had previously collected exome genotype data from the MTA sample^26^ using the Infinium^®^ HumanExome-12 v1.2 BeadChip kit (Illumina), which covers putative functional exonic variants selected from over 12,000 individual exome and whole-genome sequences. Processed and raw intensity signals for the array data can be accessed at GEO (GSE112652). SNP markers harbored at the *ADGRL3* gene were filtered in from this dataset and added to those genotyped using TaqMan^®^ assays.

### Dataset quality control and preparation for analysis

Genotype data were imported into Golden Helix® SVS 8.3.1 (Golden Helix, Bozeman, MT) for quality control analysis. Markers with a minor allele frequency (MAF) < 0.01 (rare variants), significant deviation from Hardy–Weinberg equilibrium (*P-*values < 0.0001), and a genotyping success rate < 90%, were excluded. For the Paisa and Spanish samples, a subset of variants in the *ADGRL3* minimal critical region (MCR), 5′UTR and 3′UTR were selected based on a previous ADHD association study^30^. Because the Paisa sample is a family-based cohort and recursive-partitioning analysis does not correct for kinship relatedness, only founder members from the pedigrees were included in the analyses. For the MTA sample, a total of 8568 markers with a MAF ≥ 1.0 % from the 244,414 markers genotyped with the exome chip were filtered out using linkage disequilibrium (LD) pruning, and variants within *ADGRL3* were selected for analyses. For the Kentucky sample, only four *ADGRL3* variants were selected for analyses after LD pruning of a list of markers located within the *ADGRL3* 5′UTR and MCR regions that was available to us. Variants rs7659636 and rs5010235 had been imputed from ADHD genome-wide association data funded through the Genetics Analysis Information Network (GAIN) initiative, a public-private partnership between the NIH and the private sector (https://www.genome.gov/19518664/genetic-association-information-network-gain/#al-4). *ADGRL3* variants used in this study for each cohort are presented in Supplemental Table 3.

### Advanced recursive-partitioning (tree-based) approach (ARPA)

Association studies of *ADGRL3* variants with ADHD, ODD, CD, response to stimulant treatment and severity outcome have been published elsewhere for the Paisa and Spanish populations^24,29,32,52^. We used ARPA to build a predictive framework to forecast the behavioral outcome of children with ADHD, suitable for translational applications. Our goal was to test the hypothesis that *ADGRL3* variants predisposing to ADHD also increase the risk of co-morbid disruptive symptoms, including SUD.

ARPA is a tree-based method widely used in predictive analyses because it accounts for non-linear and interaction effects, offers fast solutions to reveal hidden complex substructures and provides truly non-biased statistically significant analyses of high-dimension, seemingly unrelated data^53^. In a visionary manuscript, D.C. Rao suggested that recursive-partitioning techniques could be useful for genetic dissection of complex traits^54^. ARPA accounts for the effect of hidden interactions better than alternative methods, and is independent of the type of data (i.e., categorical, continuous, ordinal, etc.) and of the type of data distribution (i.e., fitting or not fitting normality)^54^. Furthermore, results supplied by tree-based analytics are easy to interpret visually and logically^53^. Therefore, to generate the most comprehensive and parsimonious classificatory model to predict the susceptibility to disruptive behaviors, we applied ARPA using a set of different modules implemented in the Salford Predictive Modeler^**®**^ (SPM) software, namely, Classification and Regression Trees (CART), Random Forest, and TreeNet (http://www.salford-systems.com). One important advantage of SPM when compared to other available data mining software is its ability to use raw data with sparse or empty cells, a problem frequently encountered in genetic data.

Briefly, CART is a non-parametric approach whereby a series of recursive subdivisions separate the data by dichotomization^55^. The aim is to identify, at each partition step, the best predictive variable and its best corresponding splitting value while optimizing a splitting statistical criterion, so that the dataset can be successfully split into increasingly homogeneous subgroups^55^. We used a battery of different statistical criteria as splitting rules (e.g., GINI Index, Entropy, and Twoing) to determine the splitting rule, maximally decreasing the relative cost of the tree while increasing the prediction accuracy of target variable categories^55^. The best split at each dichotomous node was chosen by either a measure of between-node dissimilarity or iterative hypothesis testing of all possible splits to find the most homogeneous split (lowest impurity). Similarly, we used a wide range of empirical probabilities (priors) to model numerous scenarios recreating the distribution of the targeted variable categories in the population^55^. Following this iterative process, each terminal node was assigned to a class outcome. To avoid finishing with an over-fitted CART predictive model (a common problem in CART analyses), and to ensure that the final splits were well substantiated, we applied tree pruning. During the procedure, predictor variables that were close competitors (surrogate predictors with comparable overall classification error to the optimal predictors) were pruned to eliminate redundant commonalities among variables, so the most parsimonious tree would have the lowest misclassification rate for an individual not included in the original data^55^.

Additionally, we applied the Random Forest (RF) methodology using a bagging strategy to exactly identify the most important set of variables predicting disruptive behaviors^56^. The RF strategy differs from CART in the use of a limited number of variables to derive each node while creating hundreds to thousands of trees. This strategy has proved to be immune to the over fitting generated by CART^56^. In RF, variables that appeared repeatedly as predictors in the trees were identified. The misclassification rate was recorded for each approach.

The TreeNet strategy was used as a complement to the CART and RF strategies because it reaches a level of accuracy that is usually not attainable by single models such as CART or by ensembles such as bagging (i.e., RF)^57^. The TreeNet algorithm generates thousands of small decision trees built in a sequential error-correcting process converging on an accurate model^57^. The number of variables considered to derive each node with RF was $\sqrt n$, where *n* is the number of independent variables (either 3 or 4).

To derive honest assessments of the derived models and have a better view of their performance on future unseen data, we applied a cross-validation strategy where both training with all the data and then indirectly testing with all the data were performed. To do so, we randomly divided the data into separate partitions (folds) of different sizes. This strategy allowed us to review the stability of results across multiple replications^55^. We used a 10-fold cross-validation as implemented in the SPM software.

A fixed-effects meta-analysis of the overall fraction of correctly classified individuals (accuracy) using the derived models from each of the four samples was applied to derive a general perspective of the SUD predictive capacity of this demographic-clinical-genetic framework.

## Results

A series of predictive models were built on our data using combinations of the following criteria: (i) the rules of splitting (GINI index, twoing, order twoing, and entropy); (ii) the priors; (iii) the size of the terminal nodes; (iv) the costs; (v) the depth of branching; and (vi) the size of the folds for cross-validation, to maximize the accuracy of the derived classification tree while considering class assignment, tree pruning, testing and cross-validation.

**Fig. 1:**
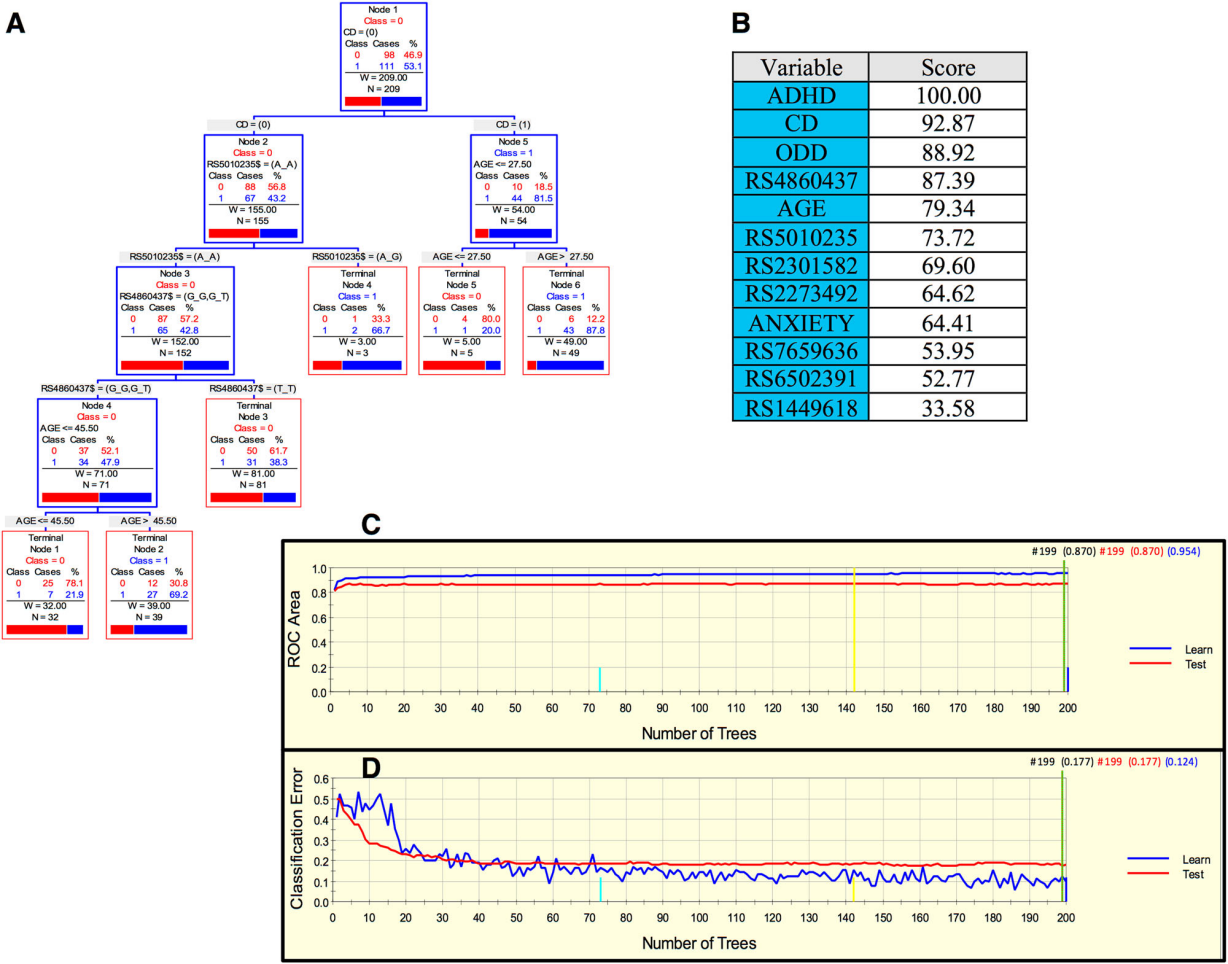
Advanced Recursive Partitioning Analysis (ARPA) for the Paisa sample. **a** Derived Classification and Regression Tree (CART) for SUD status as categorical target variable (disjunctive affection status, i.e., substance use of either alcohol, or nicotine, or other drugs). Only founder individuals were included in the analysis to avoid kinship relatedness bias. Class 0 (unaffected) is indicated in red and class 1 (affected) in blue. This derived tree for the Paisa sample included demographic (age), clinical (conduct disorder (CD)), and genetic variables (markers rs5010235 and rs4860437). The T allele of the rs4860437 variant (node 4) generates a highly discriminant split in combination with age (45.5 years) to terminal node 3 of ADHD individuals without CD (see root node 1). **b** Variable importance scores derived by Random Forest and TreeNet analysis were compatible with the variables included in the tree derived by CART. **c**, **d** TreeNet analysis to maximize the ROC area and minimize the classification error using 200 trees. The areas under the ROC curve (AUC) were 0.954 and 0.87 for learning and testing samples (blue and red curves and values, respectively), while the proportions of misclassification for SUD cases in the cross-validation experiment were 0.124 and 0.177 for learning and testing data sets, respectively

A parsimonious and informative reconstructed predictive tree derived from CART for the Paisa sample revealed demographic (age), clinical (CD), and genetic variables (rs5010235 and rs4860437) (Fig. 1a). The importance of these variables was corroborated, and their potential over fitting discarded by the TreeNet analyses that revealed a set of predictors for SUD containing those derived by CART (Fig. 1b). This predictive model displays good sensitivity and specificity as shown by areas under the receiver-operating characteristic (ROC) curve (0.954 and 0.87 for the learning and the test data, respectively) during TreeNet cross-validation using folding (Fig. 1c). The proportions of misclassification for SUD cases in the cross-validation experiment for the learning and testing data were 0.124 and 0.177, respectively (Fig. 1d).

**Fig. 2:**
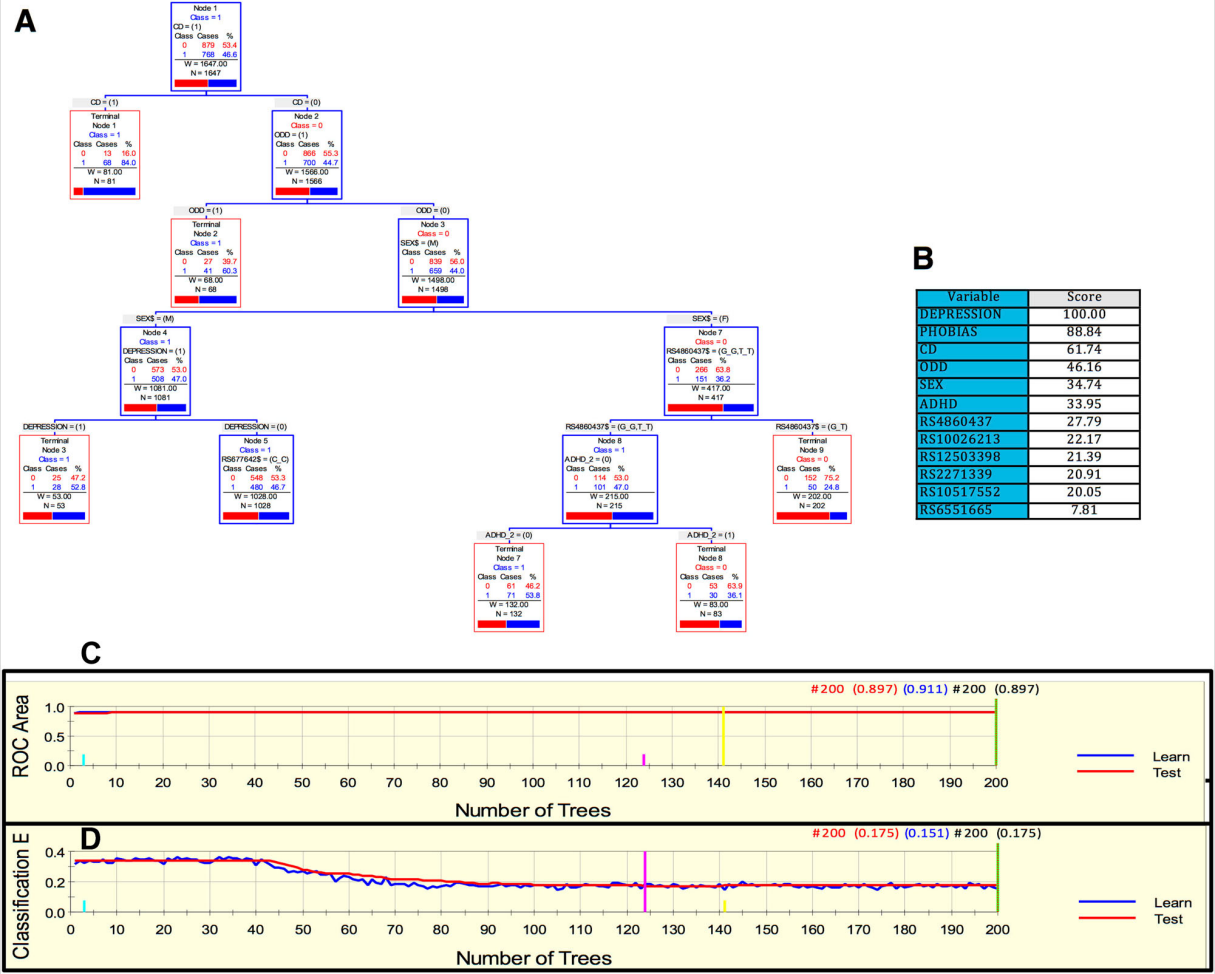
ARPA for the Spanish sample. **a** Derived tree by CART for SUD status as categorical target variable (disjunctive affection status, i.e., substance use of either alcohol, or nicotine, or other drugs). This derived tree for the Spanish sample included demographic (sex), clinical (CD, ODD, depression, and ADHD), and genetic variables (markers rs677642, rs4860437, rs1868790). **b** Variable importance scores derived by Random Forest and TreeNet analysis were compatible with the variables included in the tree derived by CART. **c**, **d** TreeNet analysis to maximize the AUC and minimize the classification error using 200 trees. The AUC were 0.911 and 0.897 for learning and testing samples while the proportions of misclassification for SUD cases in the cross-validation experiment were 0.151 and 0.175 for learning and testing data sets, respectively. Conventions as in Fig. 1

In the case of the Spanish sample, a parsimonious and informative tree was reconstructed with CART revealing demographic (sex), clinical (CD, ODD, depression, and ADHD), and genetic variables (rs4860437 and rs1868790) (Fig. 2a). The TreeNet analysis revealed a set of predictors for SUD containing those derived by CART (Fig. 2b). This predictive model displayed good sensitivity and specificity as shown by areas under the ROC curve (AUC) of 0.911 and 0.897 for learning and testing samples, respectively, during TreeNet cross-validation using folding (Fig. 2c). The proportions of misclassification for SUD cases obtained by TreeNet analysis for learning and testing data were 0.151 and 0.175, respectively (Fig. 2d).

**Fig. 3:**
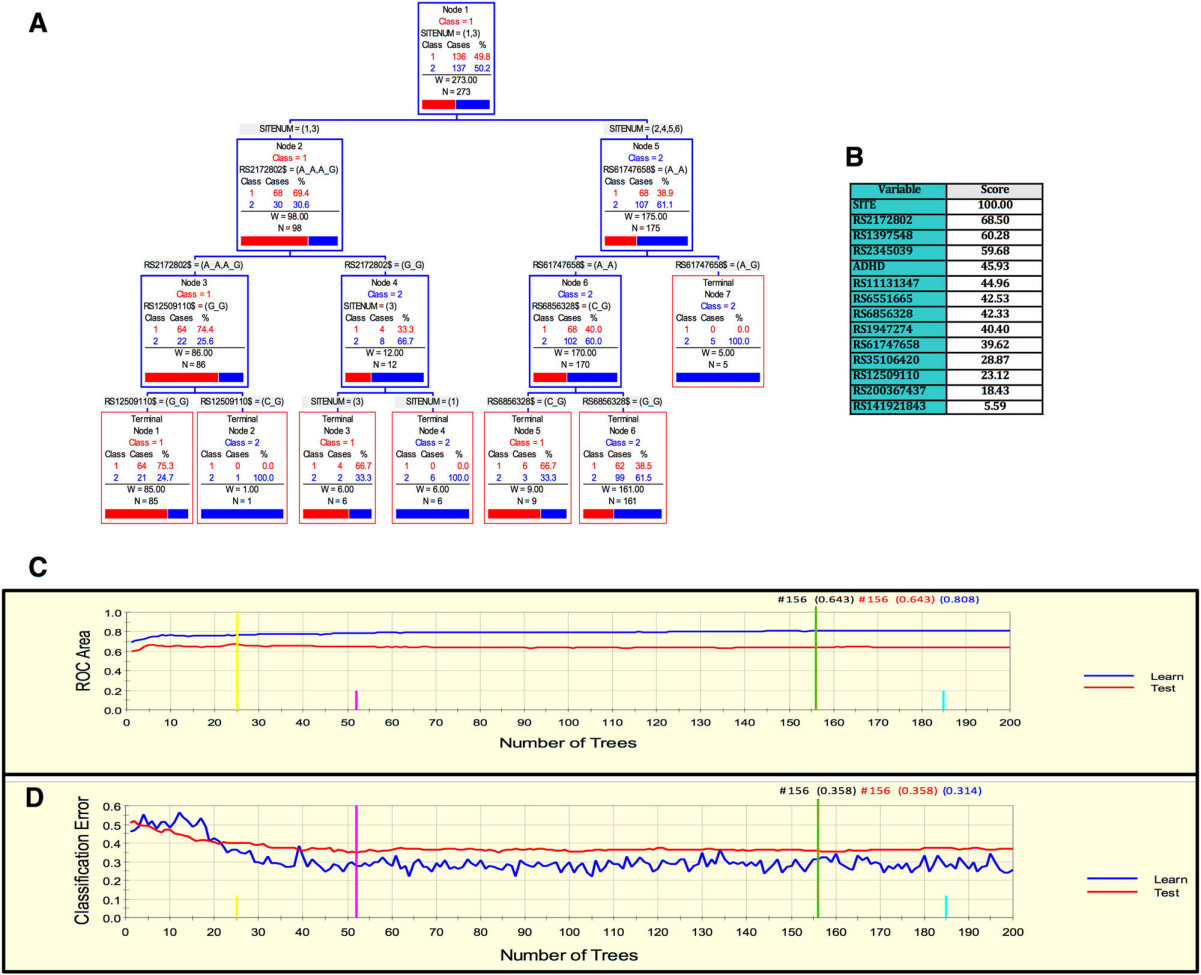
ARPA for the MTA sample. **a** Derived tree by CART for the SUD status as categorical target variable (disjunctive affection status, i.e., substance use of either alcohol, or nicotine, cannabis, or other drugs). As the MTA is a longitudinal study, we used SUD status at 96 and 120 month follow-ups and applied a lag analysis of SUD emergence. The derived tree included demographic (site of ascertainment), and genetic variables (markers rs2172802, rs61747658, rs12509110, and rs6856328). The combination of variants rs61747658 and rs2172802 generated an important discriminant splitting of SUD affected and unaffected classes. **b** Variable importance scores derived by Random Forest and TreeNet analysis were compatible with the variables included in the tree derived by CART. **c**, **d** TreeNet analysis to maximize ROC area and minimize classification error using 200 trees. The AUC were 0.808 and 0.643 for learning and testing samples, while the proportions of misclassification for SUD cases in the cross-validation experiment, for learning and testing data were 0.314 and 0.358, respectively. Conventions as in Fig. 1

As in the previous cohorts, for the MTA sample we derived a parsimonious and informative predictive tree with CART depicting demographic (site of ascertainment), and genetic variables (rs2172802, rs61747658, rs12509110, and rs6856328) (Fig. 3a). The TreeNet analyses revealed a set of predictors for SUD containing those derived by CART (Fig. 3b). This predictive model displays good sensitivity and specificity as showed by AUC of 0.808 and 0.643 for learning and testing samples, respectively, during TreeNet cross-validation using folding (Fig. 3c). The proportions of misclassification for SUD cases obtained by TreeNet analysis for learning and testing data were 0.314 and 0.358, respectively (Fig. 3d).

**Fig. 4:**
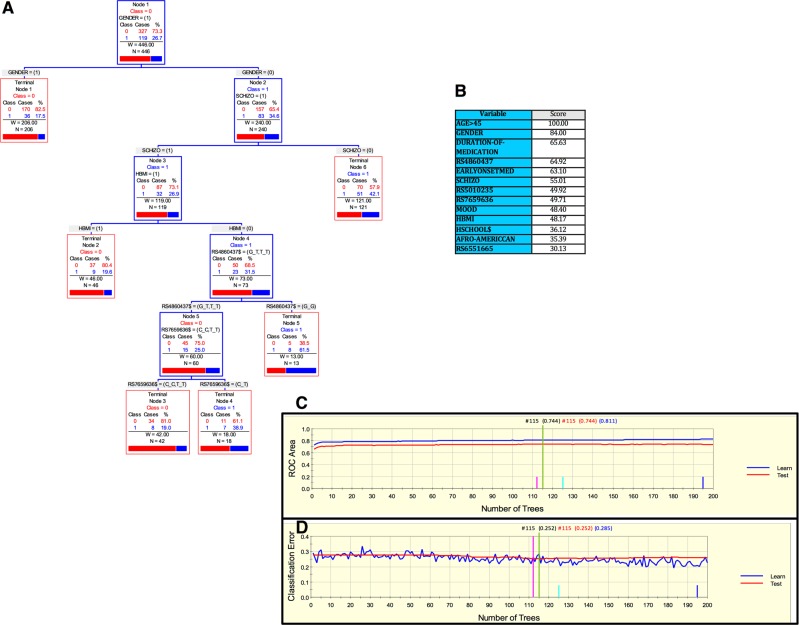
ARPA for the Kentucky sample. **a** Derived CART tree for SUD status as categorical target variable (disjunctive affection status, i.e., substance use of either alcohol, or nicotine, or other drugs). This derived tree for the Kentucky sample included demographic (sex), clinical (high Body Mass index [HBMI], and schizophrenia diagnosis), and genetic variables (markers rs4860437 and rs7659636). Notably, the T allele of the rs4860437 variant generated a split in the same direction as occurred for the derived tree in the Paisa and in the Spain samples. **b** Variable importance scores derived by Random Forest and TreeNet analysis were compatible with the variables included in the tree derived by CART. **c**, **d** TreeNet analysis to maximize ROC area and minimize classification error using 200 trees. The AUC were 0.811 and 0.744 for learning and testing samples, respectively, while the proportions of misclassification for SUD cases in the cross-validation experiment, for learning and testing data were 0.285 and 0.252, respectively. Conventions as in Fig. 1

Finally, for the Kentucky sample, we derived a parsimonious and informative predictive tree with CART involving demographic (sex), clinical (high body mass index (HBMI) and schizophrenia diagnosis), and genetic variables (rs4860437 and rs7659636) (Fig. 4a). The TreeNet analyses revealed a set of predictors for SUD containing those derived by CART (Fig. 4b). This predictive model displays good sensitivity and specificity as showed by AUC of 0.811 and 0.744 for learning and testing samples, respectively, during TreeNet cross-validation using folding (Fig. 4c). The proportions of misclassification for SUD cases obtained by TreeNet analysis for learning and testing data were 0.285 and 0.252, respectively (Fig. 4d). The results from the RF analysis were consistent with those produced by TreeNet cross-validation using folding.

**Fig. 5:**
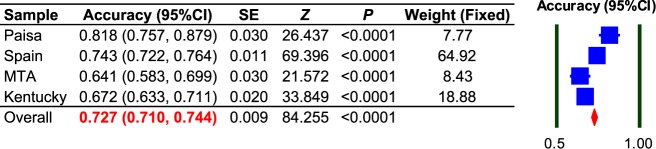
Fixed-effects meta-analysis for the prediction accuracy of the ARPA-based predictive model for SUD derived in each cohort. The overall SUD correct classification rate is ~73%. CI Confidence Interval, SE Standard Error, *Z* test statistic, *P* *P*-value

A fixed-effects meta-analysis for overall accuracy returned a value of 0.727 (95% CI = 0.710–0.744) (Fig. 5), suggesting potential eventual clinical utility of predictive values. Overall, *ADGRL3* marker rs4860437 was the most important variant predicting susceptibility to SUD, a commonality suggesting that these networks may be accurate in predicting the development of SUD based on *ADGRL3* genotypes.

We conducted independent analyses for alcohol or nicotine dependence and compared these results with those of our composite SUD phenotype, as defined by the disjunctive presence of substance use phenotypes and explained by likely common neuropathophysiological mechanisms. In general, across cohorts, we found significant alcohol and nicotine risk variants, some of which have reasonably high odd ratios (OR). For instance, in the Spain sample, marker rs2271339 conferred significant risk to nicotine use: the heterozygote genotype A/G confers 43% increased risk of being diagnosed with nicotine use (OR = 1.43, 95% CI = 1.12–1.82). In the same vein, we found in the Paisa sample that the heterozygote A/T genotype for rs1456862 confers 83% increased risk to nicotine use (corrected OR = 1.84, 95 CI% = 1.03–3.38) than the A/A genotype. Regarding alcohol use, we found in the Paisas that the heterozygote C/T genotype for rs2159140 confers susceptibility, whereas the C/C genotype does not (corrected OR = 1.64, 95 CI% = 1.01–2.72). Supplemental Fig. 1 shows the ROC curves of nicotine and alcohol use prediction in the Paisa sample. Note that the AUC is greater than 0.7 in both cases, which suggests a straight performance of markers rs1456862 and rs2159140 in predicting nicotine and alcohol use, respectively.

To determine the significance of improvement of prediction when genetic markers are introduced in the ARPA-based predictive model for SUD, we compared the performance measures (i.e., sensitivity, specificity, classification rate, and lift) across all cohorts under two disjunctive scenarios: inclusion of genetic markers or not. We found that including genetic markers improved the performance measures of the resulting ARPA-based predictive model of SUD, regardless of cohort (Supplemental Fig. 2 and Supplemental Table 1). For instance, the AUC for the Spain sample was 81.6% (95% CI = 79.8–83.4) when genetic information was included, and 77.5 (95% CI = 75.9–79.1) when it was excluded. A bootstrap-based test with 10,000 replicates revealed that the former AUC was statistically greater than the latter (*P* < 0.0001, Supplemental Table 1). Similar results were obtained for the Paisa sample: the AUC was 90% (95% CI = 86.6–93.0) when genetic information was included versus 78.8% (95% CI = 75.8–81.7) when it was not (*P* < 0.0001, Supplemental Table 1). Improvements were also observed in the correct classification rate for the Spanish and Paisa samples, the sensitivity values in all samples, the specificity in the Spanish and Paisa samples, and the lift in the Paisa sample (Supplemental Table 1). Similar results were observed for the MTA and Kentucky samples, where including genetic information in the predictive model for SUD drastically improved these performance measures (Supplemental Table 1).

## Discussion

SUD genetic epidemiological studies across multiple substances have been plagued with inconsistency in the replication of genetic association results. This may be due to reasons such as: (i) small effect size of variants expected to influence the SUD phenotype, as with any complex disease;^58^ (ii) insufficient power to detect significant associations due to small sample size;^59^ (iii) phenotypic heterogeneity of SUD across samples that may reflect different disease stages or multiple subtypes (i.e., single-drug versus poly-drug dependence/use); (iv) genetic heterogeneity arising from distinct risk genes sets; (v) ethnicity inconsistencies between discovery and replication samples;^60,61^ and (vi) comorbidity with other psychiatric conditions (e.g., ADHD) with shared genetic and environmental architecture^62,63^. Consequently, additional studies are required to identify new SUD candidate genes and to help dissect genetic contributions in the context of complex interactions with co-morbid conditions.

In this study, we present a demographic, clinical and genetic framework generated using ARPA that is able to predict the risk of developing SUD. Interestingly, marker rs4860437 showed a differential splitting pattern in the Paisa, Spain, and Kentucky cohorts. For instance, in Fig. 1a, rs4860437 splits into (G/G, G/T) and T/T; in Fig. 2a, the same variable splits into (G/G, T/T) and G/T; and in Fig. 4a, it splits into (G/T, T/T) and G/G. The most parsimonious and plausible explanation of this splitting pattern is the presence of genomic variability surrounding this proxy marker, reflecting ancestral composition. Future studies of genomic regions surrounding rs4860437 might reveal a cryptic mechanism. It is particularly compelling that *ADGRL3* marker rs4860437, which is a major predictor variable component in the trees for SUD, is in complete LD with ADHD susceptibility markers rs6551665 and rs1947274 in Caucasians^28,30,52^, suggesting that the phenotype underpinning SUD is under the pleiotropic effect of *ADGRL3* variants. Unfortunately, rs4860437 was not included in the exome chip used to genotype the MTA sample and, therefore, could not be included in the analyses for this sample. Given the limited overlap of markers across datasets and possible stratification differences among study populations, a gene- rather than a marker-level approach has been advocated^64^.

Adopting such a perspective, our results suggest that genetic variants harbored in the *ADGRL3* locus confer susceptibility to SUD in populations from disparate regions of the world. These populations are from three different countries and involve different investigators, diverse inclusion criteria, and different clinical assessments, which suggests that our results may replicate in other settings and are likely to be clinically relevant. Of particular interest is the generalization of our findings to a longitudinal study (the MTA sample), where adding genetic information to baseline data predicted the development of SUD at later ages, as determined from information gathered over a period of more than 10 years. Additionally, our results generalized to a sample of patients with severe SUD from Kentucky (U.S.) that were not ascertained on the basis of ADHD diagnosis.

The first genome-wide significant ADHD risk loci were published recently^65^. Marker rs4860437 is not represented in this dataset; however, this study was not aimed at identifying loci shared between ADHD and SUD. In any case, while genome-wide association studies are a useful tool for discovering novel risk variants—as it involves a hypothesis-free interrogation of the entire genome—the lack of genetic association may be a reflection of the polygenic, multifactorial nature of ADHD, with both common and rare variants likely contributing small effects to its etiology^66–68^. In addition, an important factor may be the genetic heterogeneity of ADHD subtypes, which may have different underlying genetic mechanisms. Therefore, genome-wide significance may identify loci with larger genetic effects, while others with smaller effects remain undetected for a given population size.

Variation in *ADGRL3* has been implicated in ADHD in diverse populations^24,27–29,31–34,69^. ADGRL3 is a member of the latrophilin subfamily of G-protein-coupled receptors (GPCR)^70^ and is most strongly expressed in brain regions implicated in the neurophysiological basis of ADHD^32,52,71^. Mouse and zebrafish knockout models also support ADGRL3 implication in ADHD pathophysiology^72,73^. More recently, Martinez et al^35^. identified a brain-specific transcriptional enhancer within *ADGRL3* that contains an ADHD risk haplotype associated with reduced *ADGRL3* mRNA expression in the thalamus. This haplotype was associated not only with ADHD, but also with disruptive behaviors, including SUD^35^. A member of the family of leucine-rich repeat transmembrane (FLRT) proteins has been identified as an endogenous postsynaptic ligand for latrophilins^74^. Interference with this interaction reduces excitatory synapse density in cultured neurons and decreases afferent input strength and dendritic spine number in dentate granule cells, which implicates ADGRL3 and FLRT3 in glutamatergic synapse development^74^. Similarly, convergent evidence from a network analysis of a gene set significantly associated and/or linked to ADHD and SUD revealed pathways involved in axon guidance, regulation of synaptic transmission, and regulation of transmission of nerve impulse^18^. These data altogether suggest that *ADGRL3* may be an important SUD susceptibility gene.

Strong evidence from clinical and genetic association studies suggests that genetic factors play a crucial role in shaping the susceptibility to both ADHD and SUD^75–81^. More strikingly, ADHD treatment has been shown to reduce the risk of SUD^82–84^. Though the neurobiological basis for this association remains unclear, a variety of causal pathways from ADHD to SUD have been proposed that involve conduct problems^77,79^. Clinical studies have suggested that the link between SUD and ADHD disappears after controlling for co-morbid CD^84–86^. In agreement with these studies, the presence of CD was a major predictor of SUD in the ARPA-based predictive models for SUD in the Paisa and Spanish cohorts (both assessed first for ADHD) (Figs. 1 and 2).

Some researchers implicate genetically mediated personality traits, such as impulsivity and lack of inhibitory control (common to ADHD and disruptive behaviors) as a link between ADHD and SUD resulting from common neurological substrates^87^. Some investigators have proposed that patients with ADHD use addictive substances to self-medicate^88^ and that the differential response to drugs of abuse and atypical behavioral regulation in response to social cues (e.g., social modeling and peer selection) may fuel substance use^77,89^. Others suggest that the poor judgment and impulsivity associated with ADHD contribute to the development of substance dependence^79^. Clinical variables from childhood have also been associated with SUD in patients with ADHD, such ADHD subtype, temper characteristics (fear, accident propensity), sexual abuse, suspension from school, and a family history of ADHD^13^.

In summary, our results support a possible functional role for *ADGRL3* in modulating drug seeking behavior. Regardless of the type of abused substance, longitudinal studies generally find that the onset of ADHD precedes that of SUD, suggesting that the psychopathology of ADHD is not secondary to SUD in most patients^79^. Accordingly, it is reasonable to consider that timely diagnosis and treatment of ADHD with stimulant medication may reduce the occurrence and/or severity of SUD. Based on the relationship with medication response^30^, we speculate that *ADGRL3* variants may underlie a differential genetic susceptibility not only to SUD, but also to the long-term protective effects of medication treatment. Confirmation of such hypothesis would have substantial public health implications. Inasmuch as ADGRL3 participates in synaptic formation and function, its involvement in SUD could be mediated by either influencing brain development or moderating drug-induced changes in synaptic strength. Molecular studies are required to elucidate the pathogenic mechanism(s) associated with ADGRL3 dysfunction in SUD.

## Supplementary information

Supplemental material
