# Supplementary material for: ADGRL3 (LPHN3) variants predict substance use disorder
Source: Transl Psychiatry. 2019 Jan 29;9:42. doi: 10.1038/s41398-019-0396-7 (PMC6351584; doi:10.1038/s41398-019-0396-7)
Supplement: Supplementary file 1 — Supplemental material [file 41398_2019_396_MOESM1_ESM.docx]

**SUPPLEMENTARY MATERIAL**

***ADGRL3* (*LPHN3*) Variants Predict Substance Use Disorder**

Mauricio Arcos-Burgos, MD PhD; Jorge I. Vélez, PhD; Ariel F. Martinez, PhD; Marta Ribasés, PhD; Josep A. Ramos-Quiroga, MD PhD; Cristina Sánchez-Mora, PhD; Vanesa Richarte, MD; Carlos Roncero, MD PhD; Bru Cormand, PhD; Noelia Fernández-Castillo, PhD; Miguel Casas, MD PhD; Francisco Lopera, MD; David A. Pineda, MD; Juan D. Palacio, MD; Johan E. Acosta-López, PhD; Martha L. Cervantes-Henriquez, MS; Manuel G. Sanchez-Rojas; Pedro J. Puentes-Rozo, PhD; Brooke S.G. Molina, PhD, and the MTA Team; Margaret T. Boden, RN; Deeann Wallis, PhD; Brett Lidbury, PhD; Saul Newman, PhD; Simon Easteal, PhD; James Swanson, PhD; Hardip Patel, PhD; Nora Volkow, MD; Maria T. Acosta, MD; Francisco X. Castellanos, MD; Jose de Leon, MD; Claudio A. Mastronardi, PhD; Maximilian Muenke, MD

**Additional evidence supporting a possible contribution of ADGRL3 to SUD pathogenicity**

Vulnerability to addictions is a complex trait with strong genetic influences that are largely shared by abusers of multiple legal and illegal addictive substances.^1^ Both linkage and association-based genome scans have been conducted for specific drug dependencies, as well as for the vulnerability to addiction across classes of substances. While each study has independently identified many different loci, these data converge to likely represent true contributions of common allelic variants to polygenic models of genetic vulnerability to polysubstance abuse.

One such locus where multiple independent studies converge is *ADGRL3* on chromosome 4. Initially, alcohol abuse was linked to chromosome 4 in the area of *ADGRL3.*^2^ This study investigated a Southwestern American Indian tribe by genome-wide linkage analysis and reported linkage to marker D4S1645. This marker is less than 300 Kb upstream of the *ADGRL3* gene. Another independent study utilized two-point linkage analysis in families with alcohol dependence to identify a nominally significant locus at D4S244.^3^ This marker is just under 2.5 Mb downstream of *ADGRL3*. While marker distance from *ADGRL3* makes this report less compelling, the two results converge to suggest that there is a locus relatively close to *ADGRL3* on chromosome 4q that influences alcohol dependence.^4,5^ Additional evidence comes from a third study that utilized whole genome association analysis to identify SNPs with significant allele frequency differences between abusers of illegal substances and control populations of both European-American and African-American descent.^6^ This study identified three SNPs within *ADGRL3* that are positive for association with a Monte Carlo *P*=0.00589. These *ADGRL3* SNPs, as well as additional *ADGRL3* SNPs have been replicated in several different populations including COGA alcohol-dependence^6,7^ and methamphetamine- dependence samples from Japan and Taiwan.^8^ In this context, our findings should encourage continued investigation on *ADGRL3*, its molecular partners and other related neurophysiological pathways in the development of SUD.

**SUPPLEMENTAL FIGURES AND TABLES**

**Supplemental Figure 1.** ROC curves predicting Nicotine and Alcohol dependency in the Paisa sample using genetic markers. AUC: area under the curve.

**Supplemental Figure 2.** ROC curves for the ARPA-based predictive model for SUD when genetic information is included versus when it is not.

**Supplemental Table 1.** Performance measures of ARPA-based predictive models of SUD for the Kentucky, MTA, Paisa and Spain samples when genetic information is included.

| **Genetics** | **Performance Measure** | | | | | ***P*** | |  |
| --- | --- | --- | --- | --- | --- | --- | --- | --- |
|  | **AUC** | **CR** | **Sensitivity** | **Specificity** | **Lift** | |  | |
| **Kentucky** |  |  |  |  |  | |  | |
| Yes | 73.2 (69.6-76.9) | 76.6 (73.0-80.1) | 91.1 (87.9-94.2) | 55.2 (48.8-61.9) | 1.26 (1.2-1.3) | | <0.0001 | |
| No | 56.1 (52.3-60.2) | 84.8 (81.8-87.8) | 14.8 (7.60-23.1) | 97.3 (95.7-98.7) | 3.29 (2.0-4.7) | |  | |
| **MTA** |  |  |  |  |  | |  | |
| Yes | 83.3 (73.0-91.9) | 85.5 (79.6-91.2) | 80.1 (60.9-95.5) | 86.4 (80.0-92.4) | 3.43 (2.6-4.8) | | <0.0001 | |
| No | 63.7 (56.0-72.0) | 79.7 (73.8-85.7) | 35.0 (20.0-51.4) | 92.3 (87.6-96.5) | 2.57 (1.7-3.6) | |  | |
| **Paisa** |  |  |  |  |  | |  | |
| Yes | 90.0 (86.6-93.0) | 89.9 (86.6-93.0) | 88.7 (83.9-93.2) | 91.3 (86.6-95.3) | 1.79 (1.6-2.0) | | <0.0001 | |
| No | 78.8 (75.8-81.7) | 82.3 (79.7-84.7) | 88.7 (86.1-91.1) | 69.0 (63.7-74.2) | 1.27 (1.2-1.3) | |  |  |
| **Spain** |  |  |  |  |  | |  | |
| Yes | 81.6 (79.8-83.4) | 80.2 (78.3-82.1) | 90.1 (87.8-92.3) | 73.1 (70.3-75.8) | 1.69 (1.6-1.8) | | <0.0001 | |
| No | 77.5 (75.9-79.1) | 76.0 (74.0-78.0) | 55.0 (51.7-58.3) | 100.0 (100.0-100.0) | 1.87 (1.8-2.0) | |  |  |

AUC: Area under the curve; CR: classification rate; *P*: *P*-value of comparing the AUC when including or not genetic information in the ARPA-based model.

**Supplemental Table 2.** Additional clinical information for the Kentucky sample.

| **Variable** | **Frequency (%)** | |
| --- | --- | --- |
|  | **No** | **Yes** |
| High BMI (> 30) | 285 (53.5) | 248 (46.5) |
| Mood | 376 (70.5) | 157 (29.5) |
| Schizophrenia | 280 (52.5) | 253 (47.5) |
| Alcohol | 191 (35.8) | 342 (64.2) |
| Drug Abuse | 386 (72.4) | 147 (27.6) |
| Smoking | 129 (24.2) | 404 (75.8) |
| Nicotine Exposure | 161 (30.2) | 372 (69.8) |
| Early-onset Medication | 251 (47.1) | 282 (52.9) |
| Duration of Medication | 98 (18.4) | 435 (81.6) |
| SUD | 81 (15.2) | 452 (84.8) |

**Supplemental Table 3.** *ADGRL3* **s**ingle-nucleotide polymorphisms (SNP) genotyped in all cohorts.

| **Population** | **SNP ID** | **Reference Allele** | **Alternate Allele** | **Variant Type** | **cDNA Position** | **Amino Acid Position** | **Amino Acid Change** | **Consequence** |
| --- | --- | --- | --- | --- | --- | --- | --- | --- |
|  |  |  |  |  |  |  |  |  |
| Paisa (n=146) | rs335271 | T | C | 5’UTR | - | - | - | - |
|  | rs335322 | G | A | intronic | - | - | - | - |
|  | rs1497917 | G | A | intronic | - | - | - | - |
|  | rs1497906 | T | A | intronic | - | - | - | - |
|  | rs1497916 | T | C | intronic | - | - | - | - |
|  | rs6851424 | G | A | intronic | - | - | - | - |
|  | rs7657344 | T | C | intronic | - | - | - | - |
|  | rs1497913 | T | C | intronic | - | - | - | - |
|  | rs7678046 | T | C | intronic | - | - | - | - |
|  | rs2345049 | T | C | intronic | - | - | - | - |
|  | rs4493573 | G | A | intronic | - | - | - | - |
|  | rs7697485 | T | C | intronic | - | - | - | - |
|  | rs11734607 | T | C | intronic | - | - | - | - |
|  | rs7695134 | T | A | intronic | - | - | - | - |
|  | rs7667328 | G | A | intronic | - | - | - | - |
|  | rs9683662 | T | C | intronic | - | - | - | - |
|  | rs6551670 | C | A | intronic | - | - | - | - |
|  | rs10027079 | T | C | intronic | - | - | - | - |
|  | rs1397548 | A | G | coding | 2984 | 937 | Pro>Pro | synonymous |
|  | rs1510921 | T | C | intronic | - | - | - | - |
|  | rs10007343 | G | A | 3’UTR | 5064 | - | - | - |
|  | rs335286 | G | A | intronic | - | - | - | - |
|  | rs186750 | G | A | intronic | - | - | - | - |
|  | rs2343249 | G | A | intronic | - | - | - | - |
|  | rs7669283 | G | A | intronic | - | - | - | - |
|  | rs10001410 | C | A | intronic | - | - | - | - |
|  | rs6551640 | T | A | intronic | - | - | - | - |
|  | rs10434218 | C | A | intronic | - | - | - | - |
|  | rs996208 | T | G | intronic | - | - | - | - |
|  | rs191915391 | G | A | coding | 644 | 157 | Ala>Ala | synonymous |
|  | rs2345047 | T | C | intronic | - | - | - | - |
|  | rs1817052 | C | A | intronic | - | - | - | - |
|  | rs2881027 | G | C | intronic | - | - | - | - |
|  | rs4860104 | G | A | intronic | - | - | - | - |
|  | rs10021694 | T | C | intronic | - | - | - | - |
|  | rs13110933 | T | C | intronic | - | - | - | - |
|  | rs2011468 | T | C | intronic | - | - | - | - |
|  | rs1355368 | G | A | intronic | - | - | - | - |
|  | rs112822655 | G | A | coding | 2522 | 783 | Thr>Thr | synonymous |
|  | rs56038622 | T | A | intronic | - | - | - | - |
|  | rs12509655 | T | A | intronic | - | - | - | - |
|  | rs10026213 | C | A | 3’UTR | - | - | - | - |
|  | rs7659636 | T | C | upstream | - | - | - | - |
|  | rs11131328 | T | A | intronic | - | - | - | - |
|  | rs6813884 | G | A | intronic | - | - | - | - |
|  | rs2047200 | G | C | intronic | - | - | - | - |
|  | rs6817476 | C | A | intronic | - | - | - | - |
|  | rs1948616 | T | C | intronic | - | - | - | - |
|  | rs17090520 | T | C | intronic | - | - | - | - |
|  | rs4860425 | C | A | intronic | - | - | - | - |
|  | rs12510774 | T | C | intronic | - | - | - | - |
|  | rs10434219 | T | C | coding | 785 | 204 | His>His | synonymous |
|  | rs12509742 | G | A | intronic | - | - | - | - |
|  | rs1450903 | G | A | intronic | - | - | - | - |
|  | rs6829758 | T | C | intronic | - | - | - | - |
|  | rs1868790 | T | A | intronic | - | - | - | - |
|  | rs6551660 | G | C | intronic | - | - | - | - |
|  | rs5010235 | G | A | intronic | - | - | - | - |
|  | rs35106420 | G | A | coding | 1567 | 465 | Arg>Gln | nonsynonymous |
|  | rs13128833 | T | A | intronic | - | - | - | - |
|  | rs995447 | T | C | intronic | - | - | - | - |
|  | rs2271339 | G | A | intronic | - | - | - | - |
|  | rs6827266 | T | G | intronic | - | - | - | - |
|  | rs7696470 | T | A | intronic | - | - | - | - |
|  | rs335288 | G | A | intronic | - | - | - | - |
|  | rs10015239 | G | A | intronic | - | - | - | - |
|  | rs11131329 | G | A | intronic | - | - | - | - |
|  | rs34586911 | G | A | coding | 353 | 60 | Arg>Arg | synonymous |
|  | rs10004368 | G | A | intronic | - | - | - | - |
|  | rs1497921 | C | A | intronic | - | - | - | - |
|  | rs1497897 | T | A | intronic | - | - | - | - |
|  | rs6843311 | T | G | intronic | - | - | - | - |
|  | rs9312082 | G | A | intronic | - | - | - | - |
|  | rs2345045 | G | C | intronic | - | - | - | - |
|  | rs10517547 | G | A | intronic | - | - | - | - |
|  | rs6846860 | T | G | intronic | - | - | - | - |
|  | rs2013374 | G | A | intronic | - | - | - | - |
|  | rs3860623 | G | A | intronic | - | - | - | - |
|  | rs11131347 | T | C | intronic | - | - | - | - |
|  | rs10517549 | T | G | intronic | - | - | - | - |
|  | rs1510925 | T | C | intronic | - | - | - | - |
|  | rs73823293 | G | A | intronic | - | - | - | - |
|  | rs10022373 | T | C | intronic | - | - | - | - |
|  | rs1032896 | T | C | intronic | - | - | - | - |
|  | rs7683260 | T | A | intronic | - | - | - | - |
|  | rs1565901 | T | C | intronic | - | - | - | - |
|  | rs1497907 | T | C | intronic | - | - | - | - |
|  | rs2172802 | G | A | intronic | - | - | - | - |
|  | rs4484334 | T | C | intronic | - | - | - | - |
|  | rs13124636 | G | A | intronic | - | - | - | - |
|  | rs1391320 | T | C | intronic | - | - | - | - |
|  | rs4283700 | T | C | intronic | - | - | - | - |
|  | rs4241640 | G | A | intronic | - | - | - | - |
|  | rs2345044 | G | A | intronic | - | - | - | - |
|  | rs7671478 | G | A | intronic | - | - | - | - |
|  | rs969965 | C | A | intronic | - | - | - | - |
|  | rs2122642 | T | C | intronic | - | - | - | - |
|  | rs6551661 | T | C | intronic | - | - | - | - |
|  | rs4860437 | T | G | intronic | - | - | - | - |
|  | rs1450900 | T | C | intronic | - | - | - | - |
|  | rs2122646 | T | C | intronic | - | - | - | - |
|  | rs1397545 | T | C | intronic | - | - | - | - |
|  | rs997407 | T | A | intronic | - | - | - | - |
|  | rs1397546 | T | C | intronic | - | - | - | - |
|  | rs4860091 | T | A | intronic | - | - | - | - |
|  | rs335307 | G | A | intronic | - | - | - | - |
|  | rs1565902 | T | C | intronic | - | - | - | - |
|  | rs958862 | G | A | intronic | - | - | - | - |
|  | rs11131334 | T | C | intronic | - | - | - | - |
|  | rs6551637 | G | A | intronic | - | - | - | - |
|  | rs11931258 | G | A | intronic | - | - | - | - |
|  | rs4552500 | G | A | intronic | - | - | - | - |
|  | rs7684100 | T | C | intronic | - | - | - | - |
|  | rs6551649 | T | C | intronic | - | - | - | - |
|  | rs2345043 | G | A | intronic | - | - | - | - |
|  | rs11723129 | T | C | intronic | - | - | - | - |
|  | rs1470721 | T | C | intronic | - | - | - | - |
|  | rs2345041 | T | C | intronic | - | - | - | - |
|  | rs17090553 | G | A | intronic | - | - | - | - |
|  | rs6551665 | G | A | intronic | - | - | - | - |
|  | rs6813183 | G | C | intronic | - | - | - | - |
|  | rs10013832 | G | A | coding | 2150 | 659 | Thr>Thr | synonymous |
|  | rs1397543 | G | A | intronic | - | - | - | - |
|  | rs1510920 | C | A | intronic | - | - | - | - |
|  | rs12507275 | T | C | intronic | - | - | - | - |
|  | rs1456862 | T | A | intronic | - | - | - | - |
|  | rs335317 | T | C | intronic | - | - | - | - |
|  | rs2132074 | G | A | intronic | - | - | - | - |
|  | rs6856328 | G | C | intronic | - | - | - | - |
|  | rs6551634 | T | A | intronic | - | - | - | - |
|  | rs1587294 | G | A | intronic | - | - | - | - |
|  | rs1542834 | G | A | intronic | - | - | - | - |
|  | rs1497909 | G | A | intronic | - | - | - | - |
|  | rs6840548 | G | A | intronic | - | - | - | - |
|  | rs12646895 | G | A | intronic | - | - | - | - |
|  | rs1901223 | G | A | intronic | - | - | - | - |
|  | rs17226265 | T | C | intronic | - | - | - | - |
|  | rs1376307 | T | C | intronic | - | - | - | - |
|  | rs990640 | T | A | intronic | - | - | - | - |
|  | rs2015569 | G | A | intronic | - | - | - | - |
|  | rs1947275 | T | C | intronic | - | - | - | - |
|  | rs6551669 | T | C | intronic | - | - | - | - |
|  | rs734644 | T | C | coding | 2252 | 693 | Asn>Asn | synonymous |
|  | rs1397547 | G | C | coding | 2882 | 903 | Arg>Arg | synonymous |
|  | rs10517552 | T | C | intronic | - | - | - | - |
|  | rs1510924 | T | G | intronic | - | - | - | - |
| MTA (n=23) | rs6856328 | G | C | intronic | - | - | - | - |
|  | rs2172802 | G | A | intronic | - | - | - | - |
|  | rs6551665 | G | A | intronic | - | - | - | - |
|  | rs35106420 | G | A | coding | 1567 | 465 | Arg>Gln | nonsynonymous |
|  | rs11131347 | T | C | intronic | - | - | - | - |
|  | rs190817915 | A | G | coding | 1323 | 384 | Val>Ile | nonsynonymous |
|  | rs1947274 | A | C | intronic | - | - | - | - |
|  | rs141921843 | C | T | coding | 1501 | 443 | Thr>Ile | nonsynonymous |
|  | rs115960764 | A | G | coding | 1659 | 496 | Glu>Lys | nonsynonymous |
|  | rs76798812 | C | T | coding | 1672 | 500 | Ala>Val | nonsynonymous |
|  | rs189474276 | C | T | coding | 1768 | 532 | Leu>Pro | nonsynonymous |
|  | rs2345039 | C | G | intronic | - | - | - | - |
|  | rs192210727 | G | T | coding | 1912 | 580 | Arg>Ile | nonsynonymous |
|  | rs61747658 | A | G | coding | 2148 | 659 | Thr>Ala | nonsynonymous |
|  | rs190183156 | A | G | coding | 2307 | 712 | Asp>Asn | nonsynonymous |
|  | rs201709145 | C | T | coding | 2521 | 783 | Thr>Met | nonsynonymous |
|  | rs12509110 | C | G | coding | 2955 | 928 | Leu>Val | nonsynonymous |
|  | rs1397548 | A | G | coding | 2984 | 937 | Pro>Pro | synonymous |
|  | rs199909144 | C | T | coding | 3049 | 959 | Phe>Ser | nonsynonymous |
|  | rs201065924 | A | G | coding | 3087 | 972 | Val>Ile | nonsynonymous |
|  | rs147399182 | A | G | coding | 3109 | 979 | Arg>His | nonsynonymous |
|  | rs200367437 | A | C | coding | 4049 | 1292 | Asn>Lys | nonsynonymous |
|  | rs73825603 | C | T | coding | 4558 | 1462 | Pro>Leu | nonsynonymous |
| Spain (n=10) | rs1587294 | G | A | intronic | - | - | - | - |
|  | rs1868790 | T | A | intronic | - | - | - | - |
|  | rs4860437 | T | G | intronic | - | - | - | - |
|  | rs6813183 | G | C | intronic | - | - | - | - |
|  | rs2271339 | G | A | intronic | - | - | - | - |
|  | rs10026213 | C | A | 3’UTR | - | - | - | - |
|  | rs10517552 | T | C | intronic | - | - | - | - |
|  | rs7667328 | G | A | intronic | - | - | - | - |
|  | rs6551665 | G | A | intronic | - | - | - | - |
|  | rs12503398 | A | G | intronic | - | - | - | - |
| Kentucky (n=4) | rs7659636 | T | C | upstream | - | - | - | - |
|  | rs5010235 | G | A | intronic | - | - | - | - |
|  | rs4860437 | T | G | intronic | - | - | - | - |
|  | rs6551665 | G | A | intronic | - | - | - | - |

**REFERENCES**

1. Arcos-Burgos M, Velez JI, Solomon BD, Muenke M. A common genetic network underlies substance use disorders and disruptive or externalizing disorders. *Hum Genet* 2012; **131:** 917-29.

2. Long JC, Knowler WC, Hanson RL, Robin RW, Urbanek M, Moore E *et al.* Evidence for genetic linkage to alcohol dependence on chromosomes 4 and 11 from an autosome-wide scan in an American Indian population. *Am J Med Genet* 1998; **81:** 216-21.

3. Reich T, Edenberg HJ, Goate A, Williams JT, Rice JP, Van Eerdewegh P *et al.* Genome-wide search for genes affecting the risk for alcohol dependence. *Am J Med Genet* 1998; **81:** 207-15.

4. Uhl GR. Molecular genetics of substance abuse vulnerability: remarkable recent convergence of genome scan results. *Ann N Y Acad Sci* 2004; **1025:** 1-13.

5. Uhl GR. Molecular genetic underpinnings of human substance abuse vulnerability: likely contributions to understanding addiction as a mnemonic process. *Neuropharmacology* 2004; **47 Suppl 1:** 140-7.

6. Johnson C, Drgon T, Liu QR, Walther D, Edenberg H, Rice J *et al.* Pooled association genome scanning for alcohol dependence using 104,268 SNPs: validation and use to identify alcoholism vulnerability loci in unrelated individuals from the collaborative study on the genetics of alcoholism. *Am J Med Genet B Neuropsychiatr Genet* 2006; **141B:** 844-53.

7. Bergen AW, Korczak JF, Weissbecker KA, Goldstein AM. A genome-wide search for loci contributing to smoking and alcoholism. *Genet Epidemiol* 1999; **17 Suppl 1:** S55-60.

8. Uhl GR, Drgon T, Liu QR, Johnson C, Walther D, Komiyama T *et al.* Genome-wide association for methamphetamine dependence: convergent results from 2 samples. *Arch Gen Psychiatry* 2008; **65:** 345-55.

9. Wilens TE, Biederman J. Alcohol, drugs, and attention-deficit/ hyperactivity disorder: a model for the study of addictions in youth. *J Psychopharmacol* 2006; **20:** 580-8.

10. Humphreys KL, Eng T, Lee SS. Stimulant medication and substance use outcomes: a meta-analysis. *JAMA Psychiatry* 2013; **70:** 740-9.
